# Podocyte NF‐*κ*B is dispensable for the pathogenesis of renal ischemia‐reperfusion injury

**DOI:** 10.14814/phy2.12912

**Published:** 2016-08-26

**Authors:** Maho Yamashita, Tadashi Yoshida, Matsuhiko Hayashi

**Affiliations:** ^1^ Apheresis and Dialysis CenterSchool of MedicineKeio UniversityTokyoJapan; ^2^ Department of General MedicineSchool of MedicineKeio UniversityTokyoJapan

**Keywords:** Acute kidney injury, ischemia‐reperfusion, NF‐*κ*B, podocytes

## Abstract

Podocytes play a central role in the formation of the glomerular filtration barrier in the kidney, and their dysfunction has been shown to result in multiple proteinuric kidney diseases. In this study, we sought to determine whether NF‐*κ*B, a proinflammatory signaling, within podocytes was involved in renal ischemia‐reperfusion (I/R) injury. Podocyte‐specific I*κ*BΔN transgenic (Pod‐I*κ*BΔN) mice, in which NF‐*κ*B was inhibited specifically in podocytes, were generated by the Cre‐loxP technology, and their phenotype was compared with control mice after bilateral renal ischemia. The effect of systemic administration of a NF‐*κ*B inhibitor, pyrrolidinedithiocarbamate (PDTC), on renal I/R injury was also examined. Pod‐I*κ*BΔN mice were phenotypically normal before surgery. Following renal I/R injury, serum concentrations of urea nitrogen and creatinine were elevated in both Pod‐I*κ*BΔN and control mice to a similar extent, whereas PDTC treatment attenuated the elevation of these parameters. Renal histological damage in I/R‐injured Pod‐I*κ*BΔN mice was also similar to I/R‐injured control mice, although it was improved by PDTC treatment. Moreover, I/R induced accumulation of inflammatory cells, such as neutrophils and macrophages, was reduced by PDTC treatment, but not by podocyte‐specific NF‐*κ*B inhibition. These results provide evidence that the NF‐*κ*B activity in podocytes does not contribute to the pathogenesis of renal I/R injury.

## Introduction

Renal ischemia‐reperfusion (I/R) injury following major surgical interventions is a leading cause of acute kidney injury (AKI) in hospitalized patients (Okusa et al. 2016). It is also the major cause of delayed graft function after cadaveric kidney transplantation. For the development of novel therapeutic approaches to prevent and/or treat these disease conditions, it is critical to identify the molecular factors and mechanisms underlying renal I/R injury.

The nuclear factor‐*κ*B (NF‐*κ*B) family of transcription factors is involved in the inflammatory process in a variety of cells (Brown et al. 1995; Traenckner et al. 1995; Sanz et al. 2010). In resting cells, NF‐*κ*B exists in the cytoplasm as an inactive dimer by binding to an inhibitory protein, I*κ*B. Upon stimulation with inflammatory signals, I*κ*B is phosphorylated on specific serine residues, serines 32 and 36, leading to its ubiquitination and consecutive proteasomal degradation. Released from I*κ*B, NF‐*κ*B is able to translocate into the nucleus, engage DNA, and initiate transcription of many genes including cytokines, chemokines, and cell adhesion molecules. Results of previous studies showed that NF‐*κ*B and its target molecules, such as monocyte chemoattractant protein‐1 and tumor necrosis factor‐*α* (TNF), were induced in rat kidneys following renal I/R injury (Donnahoo et al. 2000; Sung et al. 2002). In addition, in vivo transfection of NF‐*κ*B decoy oligonucleotides attenuated renal I/R injury in rats (Cao et al. 2004). Intravenous injection of siRNA specific for NF‐*κ*B also ameliorated renal I/R injury in mice (Feng et al. 2009). Moreover, adenovirus‐mediated overexpression of A20, a negative regulator of NF‐*κ*B, significantly reduced acute tubular necrosis and NF‐*κ*B activation in response to renal I/R injury (Lutz et al. 2008). Most recently, systemic administration of a NF‐*κ*B inhibitor, dehydroxymethylepoxyquinomicin, has been shown to ameliorate renal I/R injury in rats (Kono et al. 2013). Another NF‐*κ*B inhibitor, pyrrolidinedithiocarbamate (PDTC), has also been shown to improve folic acid‐induced AKI in mice (Kumar et al. 2015). Results of these studies suggest that activated NF‐*κ*B is a potential target for treating renal I/R injury. However, the pathogenesis of renal I/R injury is very complex and involves multiple cell types, including renal tubular epithelial cells, vascular endothelial cells, neutrophils, macrophages, and possibly podocytes (Miglio et al. 2011; Zhao et al. 2015; Okusa et al. 2016). Podocytes are required for the formation of the glomerular filtration barrier in the kidney (Reiser et al. 2010). They express a repertoire of cell‐specific proteins, such as nephrin, podocin, and synaptopodin, to retain albumin and other larger proteins in the blood. Although results of previous studies by our laboratory and others have shown that NF‐*κ*B in podocytes plays a significant role in proteinuric kidney diseases (Brähler et al. 2012; Yamashita et al. 2016), its role in renal I/R injury remains unknown.

The I*κ*BΔN mice have been developed in our laboratory (Inoue et al. 2010; Yoshida et al. 2013). They contain the human *IκBΔN* transgene separated from a universal CAG promoter by a floxed STOP sequence. Following the activation of Cre recombinase, they express I*κ*BΔN, which lacks its N‐terminal of 54 amino acids including two phosphorylation sites at serines 32 and 36, thereby continuously inhibiting NF‐*κ*B activation as a superrepressor. In this study, podocyte‐specific I*κ*BΔN transgenic (Pod‐I*κ*BΔN) mice were generated by crossing I*κ*BΔN mice with *Nphs1*‐Cre mice, which express Cre recombinase in a podocyte‐specific manner (Asano et al. 2005). By using these mice, we determined whether podocyte‐specific inhibition of NF‐*κ*B affected the severity of renal I/R injury.

## Methods

### Pod‐I*κ*BΔN mice

Animal protocols were approved by Keio University Animal Care and Use Committee. Mice used in this study were on the C57BL/6J background. Pod‐I*κ*BΔN (*Nphs1*‐Cre^+/−^/*IκBΔN* ^+/−^) mice and control (*Nphs1*‐Cre^+/−^/*IκBΔN* ^−/−^, *Nphs1*‐Cre^−/−^/*IκBΔN* ^+/−^, or *Nphs1*‐Cre^−/−^/*IκBΔN* ^−/−^) mice were generated by breeding *Nphs1*‐Cre mice (Asano et al. 2005) and I*κ*BΔN mice (Inoue et al. 2010; Yoshida et al. 2013). Genotyping was performed by PCR as described previously (Inoue et al. 2010; Yoshida et al. 2013). Four to eight mice per each genotype and per each treatment were analyzed.

### Renal ischemia‐reperfusion injury

Male Pod‐I*κ*BΔN and control mice at 12–14 weeks of age were allowed free access to water and standard mouse chow. Animals were anesthetized with an intraperitoneal injection of pentobarbital sodium (40 mg/kg). Kidneys were exposed through flank incisions. Mice were subjected to 35 min of bilateral renal ischemia or sham surgery, as previously described (Yoshida et al. 2016). Ischemia was induced by clamping both renal pedicles with nontraumatic microvessel clamps. The incisions were temporarily closed during ischemia or sham surgery. After the clamps were removed, reperfusion in the kidneys was visually confirmed. Some mice were intraperitoneally injected with 200 mg/kg PDTC (Sigma‐Aldrich, St. Louis, MO) 3 h before surgery. A duration of 24 or 72 h after reperfusion, the mice were killed under pentobarbital anesthesia, and blood samples as well as the kidneys were harvested. Kidneys were divided into multiple pieces for histological analyses and total RNA extraction.

### Serum urea nitrogen and creatinine

Serum concentrations of urea nitrogen were determined by the urease‐indophenol method (Wako Pure Chemical, Osaka, Japan). Serum creatinine concentrations were measured by an enzymatic method (Wako Pure Chemical).

### Histology and Immunostaining

The kidneys were fixed in 4% paraformaldehyde and embedded into paraffin. Sections (5‐*μ*m) were prepared and subjected to hematoxylin–eosin staining and immunohistochemistry. Histological analyses were performed in a blind manner using an arbitrary scale, as described previously (Homma et al. 2014; Yoshida et al. 2016). Proteinaceous casts and tubular necrosis were graded as follows: 0 (no damage), 1 (patchy isolated damage), 2 (damage less than 25%), 3 (damage between 25% and 50%), and 4 (more than 50% damage). Immunohistochemistry was performed with antibodies for neutrophil (7/4; Abcam, Cambridge, MA) and F4/80 (CI:A3‐1; Abcam), as described previously (Yoshida et al. 2008, 2016; Yamashita et al. 2016). Staining was visualized by diaminobenzidine, and sections were counterstained by hematoxylin.

### RNA extraction and real‐time RT‐PCR

Total RNA was extracted, and real‐time RT‐PCR was performed as described previously (Yoshida et al. 2008). Primer sequences were as follows: NGAL‐F: 5′‐AACATTTGTTCCAAGCTCCAGGGC‐3′ and NGAL‐R: 5′‐CAAAGCGGGTGAAACGTTCCTTCA‐3′.

### Primary culture of podocytes

Primary culture of murine podocytes was performed as described previously (Yamashita et al. 2016). Cultured podocytes derived from Pod‐I*κ*BΔN mice and control mice, respectively, were treated with 10 ng/mL TNF for 24 h, and subjected to immunofluorescence studies with antibodies for p65 (F6; Santa Cruz Biotechnology, Santa Cruz, CA) and podocin (Abcam).

### Statistical analyses

**Figure 1:**
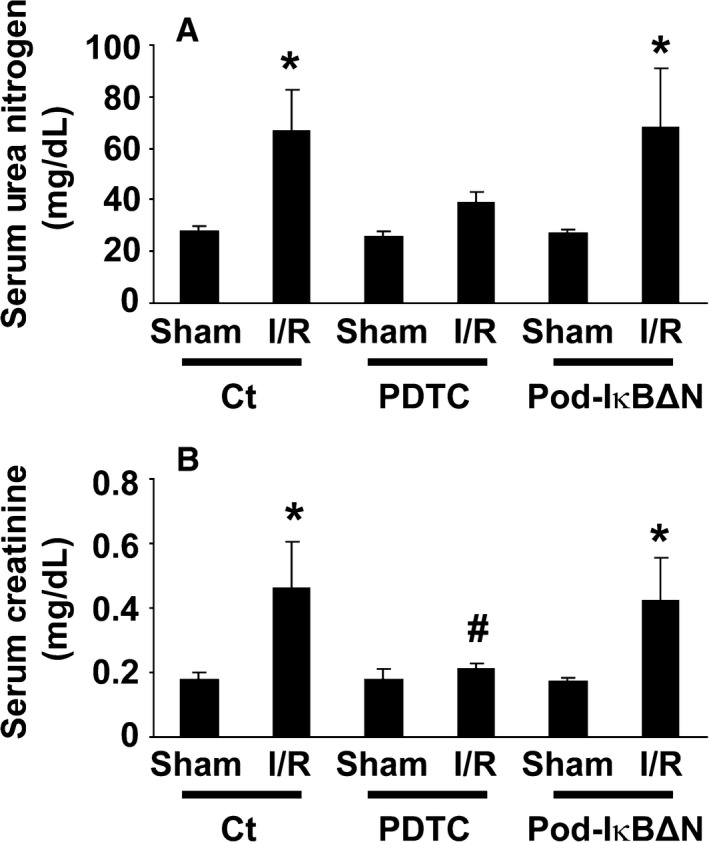
The NF‐*κ*B inhibitor, pyrrolidinedithiocarbamate (PDTC), but not podocyte‐specific NF‐*κ*B inhibition, attenuated renal ischemia‐reperfusion (I/R) injury. Pod‐I*κ*BΔN and control mice were subjected to bilateral renal ischemia for 35 min (I/R) or sham‐operation. A subset of mice were treated with 200 mg/kg PDTC intraperitoneally 3 h before I/R. Serum levels of urea nitrogen (A) and creatinine (B) were measured 24 h after reperfusion. *n* = 5–8 per each group. *P < 0.05 compared with sham‐operated mice. #*P* < 0.05 compared with I/R‐injured control mice.

**Figure 2:**
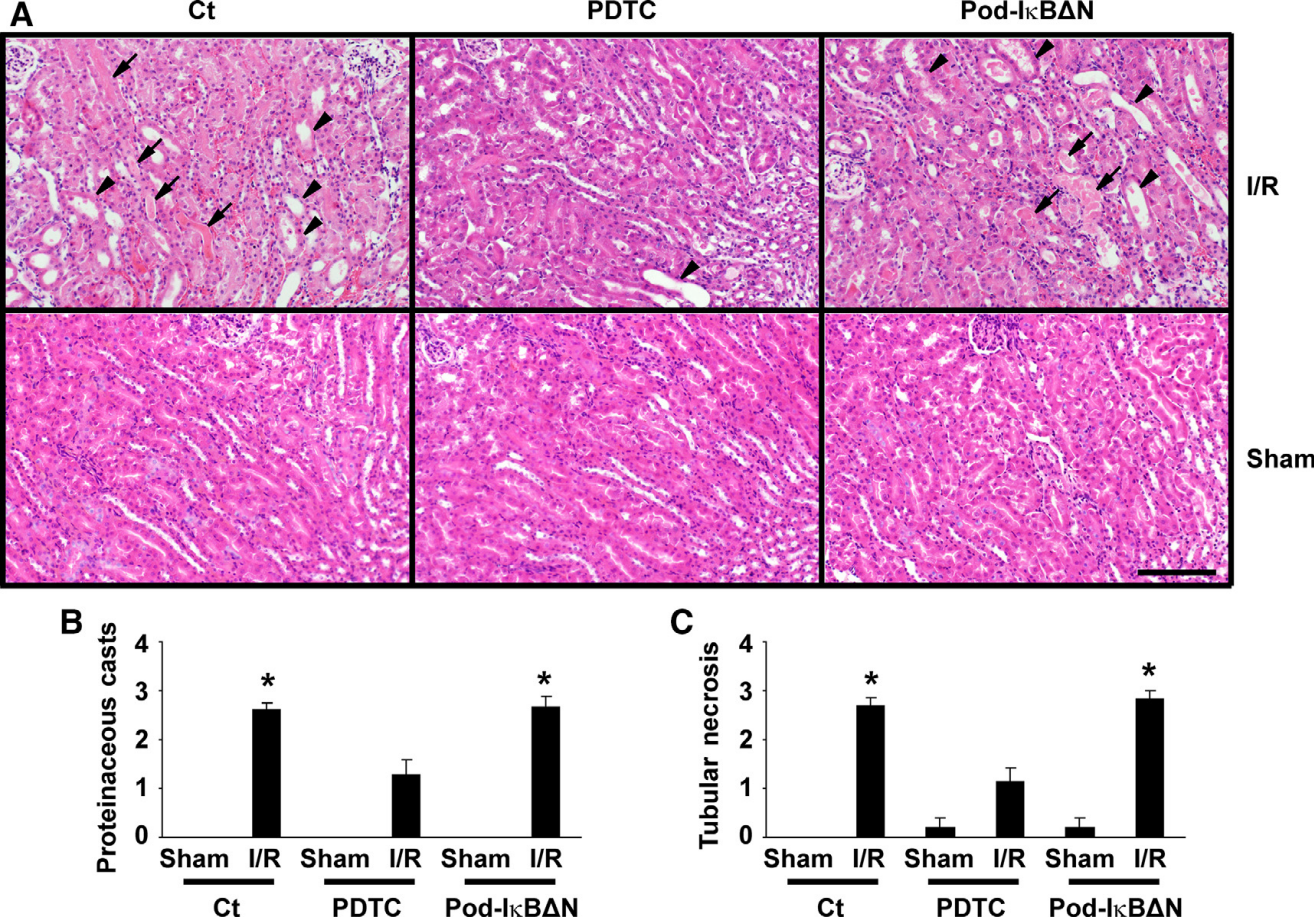
Pyrrolidinedithiocarbamate (PDTC), but not podocyte‐specific NF‐*κ*B inhibition, improved renal histological damage following ischemia‐reperfusion (I/R) injury. Pod‐I*κ*BΔN and control mice were subjected to bilateral renal ischemia for 35 min (I/R) or sham‐operation. A subset of mice were treated with 200 mg/kg PDTC intraperitoneally 3 h before I/R. Renal histology was examined 24 h after reperfusion. (A) Representative pictures of hematoxylin–eosin staining are shown. Bar: 100 *μ*m. Arrows indicate intratubular casts. Arrowheads indicate tubular necrosis. B, C: Levels of the formation of proteinaceous casts (B) and tubular necrosis (C) were scored semiquantitatively. *n* = 5–8 per each group. *P < 0.05 compared with sham‐operated mice.

Data are presented as mean ± SEM. Statistical analyses were done by SigmaPlot/SigmaStat9 (Systat Software Inc, San Jose, CA). After confirming that the data passed the normality test for parametric analyses, one‐way factorial ANOVA with a post hoc Fisher protected least significant difference test was performed (Figs. 1A,B, 4, 5C,D, and 6A). Nonparametric Kruskal–Wallis test was also performed (Figs. 2B,C, and 6B). *P* values < 0.05 were considered significant.

## Results

### A NF‐*κ*B inhibitor, PDTC, attenuated renal I/R injury in mice

Results of the previous studies showed that systemic administration of a NF‐*κ*B inhibitor, dehydroxymethylepoxyquinomicin, ameliorated renal I/R injury in rats (Kono et al. 2013). To confirm and extend these results, we first examined the effect of PDTC, another NF‐*κ*B inhibitor, on renal I/R injury. Male control mice at 12–14 weeks of age were intraperitoneally injected with PDTC, and then received bilateral I/R injury for 35 min. A duration of 24 h after reperfusion, serum levels of urea nitrogen significantly increased in control mice (66 ± 16 mg/dL), compared with sham‐operated mice (28 ± 2 mg/dL) (Fig. 1A). PDTC treatment attenuated the elevation of serum urea nitrogen following I/R injury (39 ± 4 mg/dL). Serum concentrations of creatinine exhibited a similar trend (Fig. 1B). Histological analyses revealed that PDTC treatment significantly improved the formation of proteinaceous casts and tubular necrosis, two histological features of renal I/R injury (Fig. 2). These results suggest that the systemic blockade of the NF‐*κ*B activity attenuates renal I/R injury.

### Podocyte‐specific NF‐*κ*B inhibition did not improve renal I/R injury

**Figure 3:**
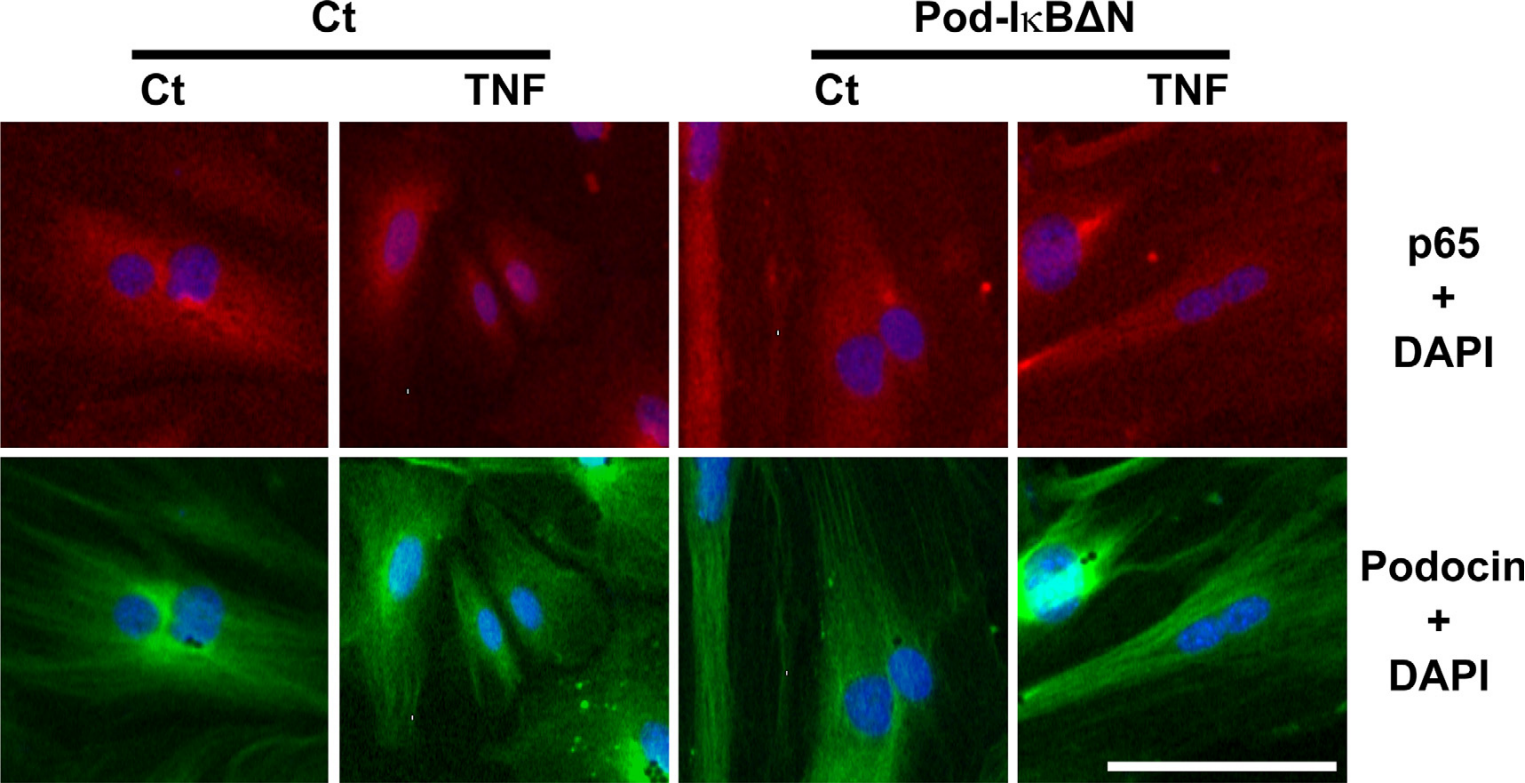
Tumor necrosis factor (TNF) induced p65 translocation from the cytoplasm to the nucleus in cultured podocytes derived from control mice, but not from Pod‐I*κ*BΔN mice. Primary cultures of podocytes derived from Pod‐I*κ*BΔN and control mice, respectively, were treated with TNF for 24 h. Expression of p65 (Red) as well as podocin (Green) was examined by immunofluorescence studies. Nuclear staining was performed with 4′,6‐diamidino‐2‐phenylindole (DAPI; Blue). Bar: 100 *μ*m.

To determine the cell‐autonomous role of the NF‐*κ*B signaling in podocytes for renal I/R injury, we utilized Pod‐I*κ*BΔN mice, in which NF‐*κ*B was inhibited specifically in the podocytes. Results of our previous studies showed that Pod‐I*κ*BΔN mice were phenotypically normal at the physiological conditions, and that the amount of proteinuria was significantly lower in Pod‐I*κ*BΔN mice than control mice in adriamycin‐induced nephropathy (Yamashita et al. 2016). In this study, we first examined the localization of p65 in cultured podocytes derived from Pod‐I*κ*BΔN mice and control mice, respectively. Results showed that p65 was retained in the cytoplasm following TNF treatment in cultured podocytes derived from Pod‐I*κ*BΔN mice, whereas it was translocated into the nucleus in response to TNF in cultured podocytes derived from control mice (Fig. 3). These results suggest that the NF‐*κ*B activity is selectively inhibited in podocytes in Pod‐I*κ*BΔN mice.

**Figure 4:**
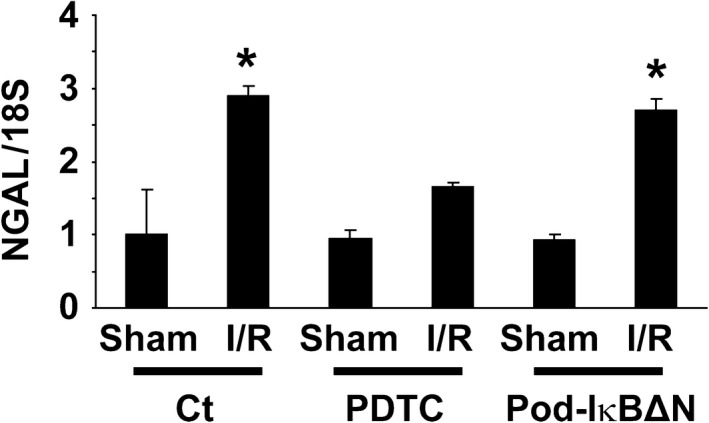
Pyrrolidinedithiocarbamate (PDTC), but not podocyte‐specific NF‐*κ*B inhibition, attenuated ischemia‐reperfusion (I/R) injury induced increases in NGAL expression. Pod‐I*κ*BΔN and control mice were subjected to bilateral renal ischemia for 35 min (I/R) or sham‐operation. A subset of mice were treated with 200 mg/kg PDTC intraperitoneally 3 h before I/R. A duration of 24 h after reperfusion, renal NGAL expression was examined by real‐time RT‐PCR. *n* = 5–8 per each group. *P < 0.05 compared with sham‐operated mice.

Pod‐I*κ*BΔN and control mice were subjected to renal I/R injury. As shown in Figure 1, serum concentrations of urea nitrogen as well as creatinine did not differ between Pod‐I*κ*BΔN and control mice following I/R injury. I/R‐induced histological damage was also similar between Pod‐I*κ*BΔN and control mice (Fig. 2). Moreover, I/R injury‐induced increases in expression of NGAL, a marker of AKI, were similar between Pod‐I*κ*BΔN and control mice, whereas PDTC treatment attenuated I/R injury‐induced increase in NGAL expression (Fig. 4). These results suggest that the NF‐*κ*B signaling in podocytes does not contribute to renal I/R injury.

### I/R induced accumulation of inflammatory cells was reduced by PDTC treatment, but not by podocyte‐specific NF‐*κ*B inhibition

**Figure 5:**
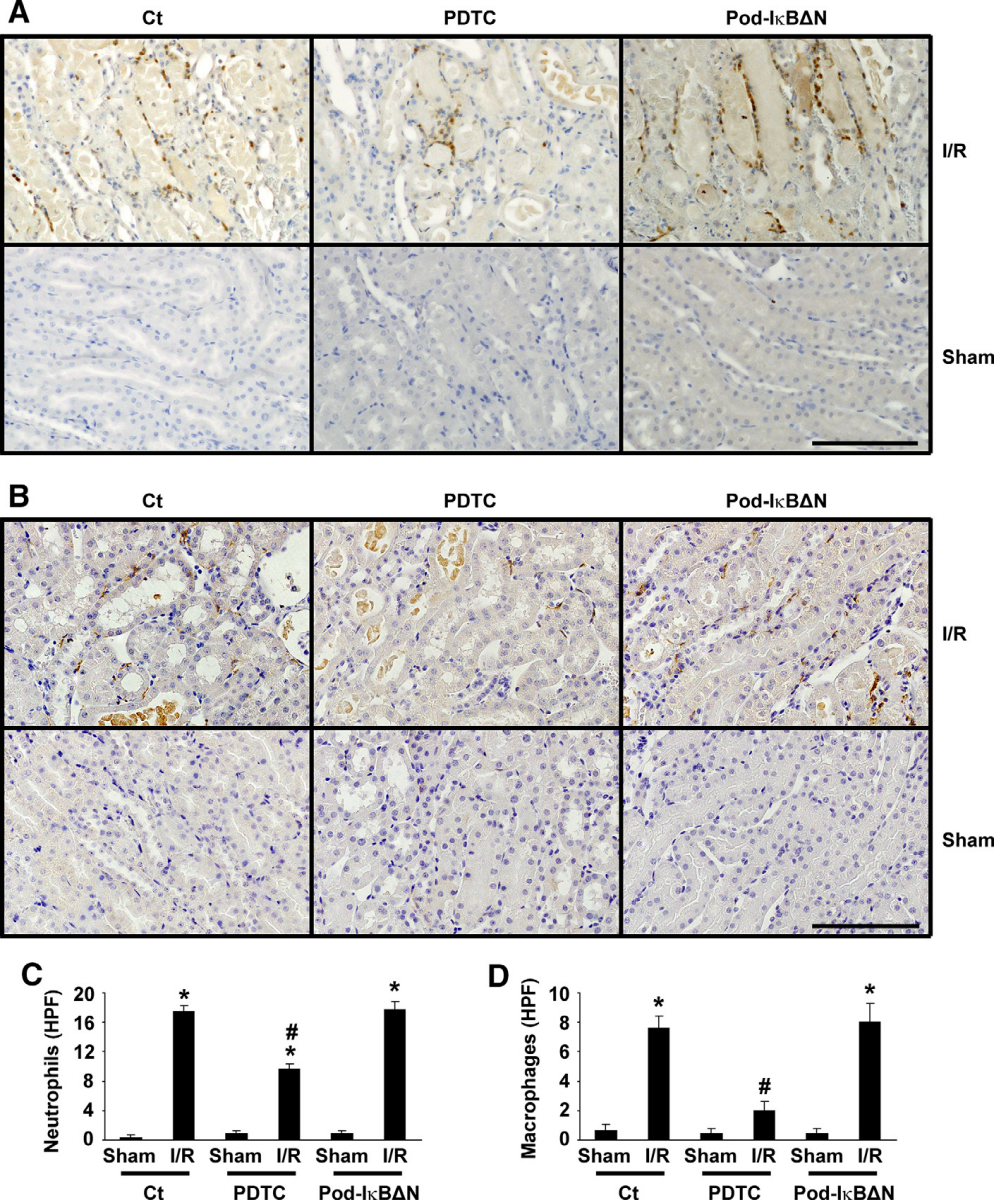
Ischemia‐reperfusion (I/R) induced accumulation of inflammatory cells was reduced by pyrrolidinedithiocarbamate (PDTC), but not by podocyte‐specific NF‐*κ*B inhibition. Pod‐I*κ*BΔN and control mice were subjected to bilateral renal ischemia for 35 min (I/R) or sham‐operation. A subset of mice were treated with 200 mg/kg PDTC intraperitoneally 3 h before I/R. Accumulation of neutrophils (A and C) and macrophages (B and D) was examined 24 h after reperfusion. *n* = 5–7 per each group. A and B: Representative pictures of immunohistochemical staining for neutrophils (A) and macrophages (B) are shown. Neutrophils (A) and macrophages (B) were visualized by diaminobenzidine, and sections were counterstained with hematoxylin. Bars: 100 *μ*m. C and D: The numbers of neutrophils (C) and macrophages (D) per five random fields in the kidneys were quantified. *P < 0.05 compared with sham‐operated mice. #*P* < 0.05 compared with I/R‐injured control mice.

I/R injury has been shown to induce the infiltration of neutrophils and macrophages in the kidneys. Accumulation of these inflammatory cells was examined by immunohistochemistry. Results showed that both neutrophils and macrophages increased following renal I/R injury (Fig. 5). Although PDTC treatment reduced the number of these inflammatory cells in the kidneys, I/R induced accumulation of these cells did not differ between Pod‐I*κ*BΔN and control mice (Fig. 5). These results suggest that podocyte NF‐*κ*B does not play a significant role in the infiltration of inflammatory cells during renal I/R injury.

### Podocyte NF‐*κ*B also did not contribute to renal damage at later stage of AKI

**Figure 6:**
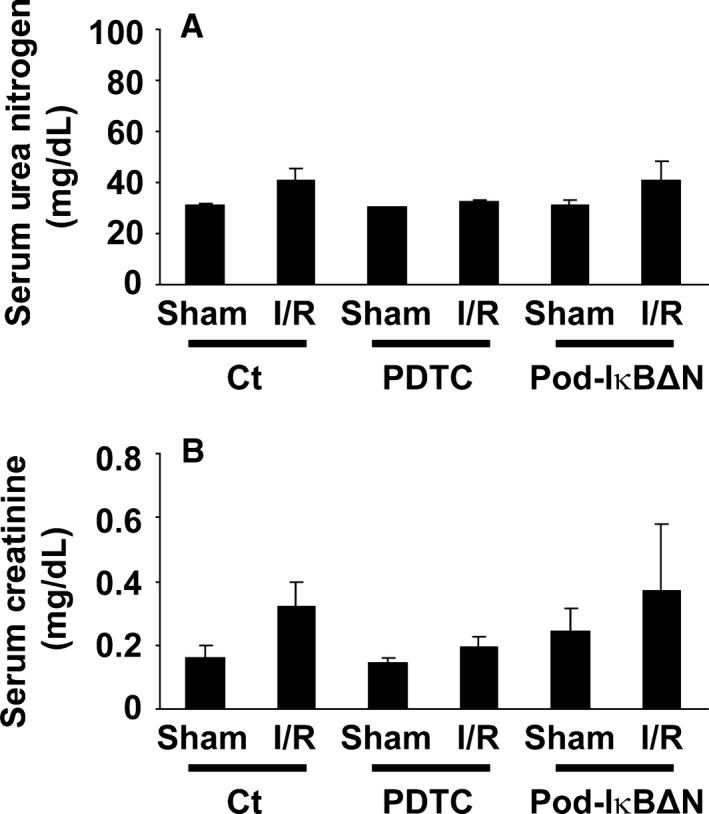
Renal ischemia‐reperfusion (I/R) injury was modest at later stage of AKI. Pod‐I*κ*BΔN and control mice were subjected to bilateral renal ischemia for 35 min (I/R) or sham‐operation. A subset of mice were treated with 200 mg/kg pyrrolidinedithiocarbamate (PDTC) intraperitoneally 3 h before I/R. Serum levels of urea nitrogen (A) and creatinine (B) were measured 72 h after reperfusion. *n* = 4 per each group.

The effect of podocyte‐specific NF‐*κ*B inhibition on later stage of AKI was also examined. A duration of 72 h after reperfusion, I/R injury induced increases in serum concentrations of urea nitrogen and creatinine were modest in both Pod‐I*κ*BΔN and control mice (Fig. 6). These results do not suggest that the contribution of podocyte NF‐*κ*B is different between early and late stages of AKI.

## Discussion

Results of the previous studies showed that the NF‐*κ*B signaling in various renal cells contributed to multiple renal diseases. For example, adenovirus‐mediated overexpression of I*κ*BΔN in renal tubular cells prevented tubulointerstitial injury in protein‐overloaded rats (Takase et al. 2003). Endothelial cell‐specific overexpression of I*κ*BΔN attenuated hypertension‐induced albuminuria and renal damage in mice (Henke et al. 2007). Fibroblast‐specific inhibition of NF‐*κ*B by I*κ*BΔN transgene attenuated renal fibrosis in a unilateral ureteral obstruction model (Inoue et al. 2010). Moreover, we previously showed that podocyte‐specific inhibition of NF‐*κ*B attenuated proteinuria in adriamycin‐induced nephropathy in mice (Yamashita et al. 2016). The effect of podocyte‐specific NF‐*κ*B inhibition on proteinuric kidney disease was also examined by the deletion of NF‐*κ*B essential modulator (NEMO), a subunit of the I*κ*B kinase complex required for phosphorylation and proteasomal degradation of I*κ*B (Brähler et al. 2012). The NEMO deletion in the podocytes reduced proteinuria in nephrotoxic sheep serum‐induced glomerulonephritis in mice (Brähler et al. 2012). Although the aforementioned studies provide evidence that the NF‐*κ*B signaling in multiple renal cells play an important role in the pathogenesis of various renal disease conditions, results of this study demonstrate that the NF‐*κ*B activity in podocytes does not contribute to renal I/R injury. Because systemic inhibition of NF‐*κ*B has been shown to improve renal I/R injury (Cao et al. 2004; Lutz et al. 2008; Feng et al. 2009; Kono et al. 2013), the NF‐*κ*B activity in other renal cell types except podocytes is likely to be involved in the pathogenesis of I/R injury. In future, it is required to determine the effect of deletion of the NF‐*κ*B activity in proximal tubular cells, distal tubular cells, endothelial cells, fibroblasts, and immune cells, serially or in combination, on renal I/R injury using genetically modified mouse models. In addition, it should be noted that systemic NF‐*κ*B inhibitors, including PDTC, have been shown to block other intracellular signaling pathways, and therefore alter the levels of the oxidative stress and nitric oxide generation (Tapia et al. 2008; Tugcu et al. 2008). It should be careful to interpret the data using these compounds. It is hoped that specific NF‐*κ*B inhibitors are developed in future.

Although this study does not provide evidence for the involvement of NF‐*κ*B within podocytes in renal I/R injury, podocytes per se have been shown to participate in the pathogenesis of renal I/R injury. For example, results of the previous studies showed that renal I/R injury induced structural damage to the integrity of podocytes, as assessed by the electron micrography (Zhao et al. 2015). They also showed that the structural changes in podocytes were accompanied by the upregulation of TRPC6 expression. It is possible that the induction of TRPC6 and subsequent Ca^2+^ influx, rather than the activation of NF‐*κ*B, are the main signaling pathway for I/R‐induced damage in podocytes. Moreover, using cultured human podocytes, peroxisome proliferator‐activated receptor agonists were shown to prevent apoptotic cell death induced by oxygen/glucose deprivation‐reoxygenation, an in vitro model of renal I/R injury (Miglio et al. 2011). Although the results of this study showed that the NF‐*κ*B signaling in podocytes does not play a significant role in renal I/R injury, they do not contradict these previous studies. Podocytes are still one of the possible cellular targets for the treatment of renal I/R injury.

In summary, the results of this study provide evidence that the NF‐*κ*B signaling in podocytes does not contributes to renal I/R injury. Further studies are needed to identify the renal cell types where NF‐*κ*B plays a key role in this disease condition.

## Conflict of Interest

None declared.
